# Value-Added Application of Waste Rubber and Waste Plastic in Asphalt Binder as a Multifunctional Additive

**DOI:** 10.3390/ma12081280

**Published:** 2019-04-18

**Authors:** Tianqing Ling, Ya Lu, Zeyu Zhang, Chuanqiang Li, Markus Oeser

**Affiliations:** 1 School of Architecture and Urban planning, Chongqing Jiaotong University, Chongqing 400074, China; lingtq@163.com; 2 School of Civil Engineering, Chongqing Jiaotong University, Chongqing 400074, China; 3 Institute of Highway Engineering, RWTH Aachen University, 52074 Aachen, Germany; 4 College of Materials Science and Engineering, Chongqing Jiaotong University, Chongqing 400074, China; lichuanqiang_cn@163.com; 5 Institute of Highway Engineering, RWTH Aachen University, 52074 Aachen, Germany; oeser@isac.rwth-aachen.de

**Keywords:** waste rubber, waste plastic, asphalt binder, warm mix, rheological property

## Abstract

The feasibility and effectivity of recycling waste rubber and waste plastic (WRP) into asphalt binder as a waste treatment approach has been documented. However, directly blending WRP with asphalt binder brings secondary environmental pollution. Recent research has shown that the addition of WRP into asphalt binder may potentially improve the workability of asphalt binder without significantly compromising its mechanical properties. This study evaluates the feasibility of using the additives derived from WRP as a multifunctional additive which improves both the workability and mechanical properties of asphalt binder. For this purpose, WRP-derived additives were prepared in laboratory. Then, three empirical characteristics—viscosity, rutting factor, fatigue life were analyzed. Fourier transform infrared spectroscopy (FTIR) and differential scanning calorimetry (DSC) were employed to evaluate the effect of WRP-derived additive on the workability and chemical and mechanical properties of base binder. The dispersity of WRP-derived additive inside asphalt binder was also characterized using fluorescence microscope (FM). Results from this study showed that adding WRP-derived additive increases the workability of base binder. The WRP-derived additive appears positive on the high- and low- temperature performance as well as the fatigue life of base binder. The distribution of the WRP-derived additive inside base binder was uniform. In addition, the modification mechanism of WRP-derived additive was also proposed in this paper.

## 1. Introduction

The extensive use of rubber and plastic creates more convenient lives for citizens. However, the waste treatment department is under high pressure due to the rapid increase of waste rubber and waste plastic (WRP) [1]. Conventionally, the WRP are treated through landfilling or burning, although these methods result in new environmental concerns, e.g., air pollution, dioxin emissions, land pollution, and even ocean pollution [1,2,3,4,5,6,7]. Asphalt binder is commonly used in pavement industry around the world as a paving material because of its merits such as easy construction and driving comfort [8,9,10,11,12,13,14,15]. By means of incorporating wastes into asphalt binder, a large amount of domestic garbage or industrial refuse can be consumed with environmental benefits [1,16,17,18,19]. Thus, attention has been focused on the possibility of using asphalt binder as an innovative waste treatment method [20,21,22]. Specifically, previous studies have demonstrated the feasibility of recycling various types of waste materials into asphalt materials, for instance, end-of-life vehicle tires [23], waste plastic [2,24], electronic waste powders [17], marble waste [25], waste bleaching clays [26], waste glass powder [27], waste wood resources [28], etc.

During the late 1980s and early 1990s, highway industry was encouraged by the US Department of Transportation (USDOT) and Federal Highway Administration (FHWA) to utilize recycled crumb rubber (CR) in highway construction [29,30]. Numerous studies focusing on CR modified asphalt binder were then carried out. Previous studies reported that CR significantly increase the mechanical properties of asphalt binder [31,32,33]. By applying CR powders, both the high and low temperature performance of asphalt binder can be significantly enhanced [22,33,34]. Specifically, CR increases the viscosity and rutting resistance of base binder at high temperatures as well as the creep compliance at low temperatures [35]. The fracture resistance property of CR modified asphalt binder (CRMA) was also proved to be much better than that of base binder [36,37]. Numerous efforts were also spent to dig into the modification mechanism. It is believed that the swelling and dissolution occurring between rubber particle and asphalt binder play a fundamental role in increasing binder’s viscosity [31,38,39,40,41].

Inspired by the success of the value-added application of waste rubber, asphalt engineers and researchers put their eyes on waste plastic which is another source of pollution that is difficult to manage [42]. Like waste rubber, the feasibility of applying waste plastic into asphalt pavement as a modifier was also demonstrated [2,24,43]. Previous studies revealed that waste plastic can be an alternative for the current binder modifiers. It was reported that waste plastic can improve the rutting resistance, fracture resistance, thermal stability, degradation, and low temperature cracking properties of base binder [44,45]. Waste plastic was demonstrated to have the potential to perform as an anti-aging and anti-striping agent in base binder [46,47]. 

However, recycling WRP into asphalt raises the environmental concerns about emissions generated during mixing and paving processes of asphalt mixture. This is because WRP improves the viscosity of base binder which in turn results in higher manufacturing and paving temperatures of the asphalt mixture [48]. Furthermore, poor workability caused by binder’s high viscosity becomes another hurdle to promote the application of WRP modified asphalt binder [23]. One feasible approach to handle the abovementioned issue is integrating the WPR additive with warm mix technologies [49,50]. Previous studies documented that by combining warm mix technology, WRP can be effectively consumed in asphalt binder with enhanced mechanical properties as well as environmental benefits. By incorporating warm mix technologies, the manufacturing and paving temperature of WRP modified asphalt mixture can be reduced by around 20 °C [51,52]. Based on the mechanism, the warm mix technologies can be grouped into three categories: foaming technologies, addition of chemical additives, and addition of organic additives [53]. Aspha-min, Evotherm, and Sasobit are the corresponding represent commercial products, respectively. The organic additives added into base binder are waxes. When the binder’s temperature increases above the melting point of wax, the viscosity of the binder decreases. Nevertheless, it should be considered that the use of warm mix technologies creates extra cost for pavement construction. Therefore, producing a modifier derived from WRP with the capacity for increasing service property and workability of asphalt binder would be a win-win solution for the abovementioned issues.

Chemically, rubber and plastic are both high-molecular polymers which can be thermally cracked into small-molecules. Rubber can be thermally cracked into pyrolysis gas, pyrolysis oil, and heavy lysate [54,55]. Plastic can be thermally cracked into wax [56]. And the components of these pyrolysis depend on the thermal cracking condition. Thus, by choosing suitable thermal cracking conditions, it is possible to produce an asphalt binder modifier derived from WRP for the purpose of increasing both workability and mechanical properties of asphalt binder.

The objective of this study is to evaluate the effect of a binder additive, which is produced by thermal cracking WRP in laboratory, on mechanical and chemical properties of base binder. To this end, three empirical characteristics—namely, penetration, softening point, and ductility—were measured. Rheological properties including viscosity, rutting factor, fatigue life, and bending stiffness were characterized. The effect of thermally cracked WRP (CWRP) on chemical properties of asphalt binder was also investigated with Fourier transform infrared spectroscopy (FTIR) and differential scanning calorimetry (DSC). The dispersity of CWRP inside asphalt binder was evaluated using fluorescence microscope (FM).

## 2. Materials and Experimental Program

### 2.1. Materials

In this study, asphalt binder with a penetration grade of 70 (Pen 70) was used as the base binder. The asphalt binder used in this study is supplied by Shell Co., Ltd. (Foshan, China). Its properties are presented in Table 1. 

**Figure 1:**
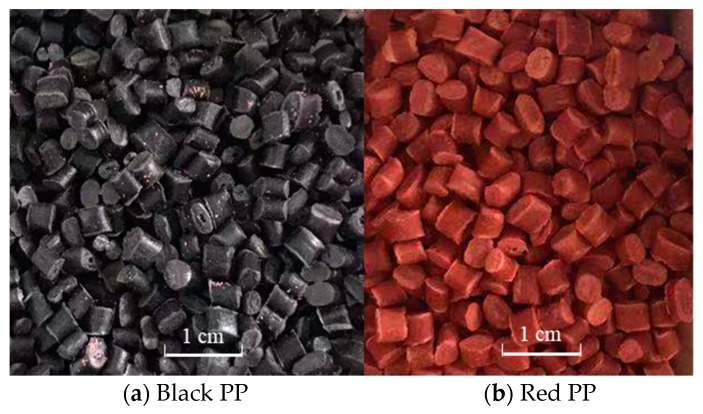
Waste plastic particles with different colors: (**a**) black and (**b**) red.

WRP particles used in this study were provided by two plants in Chongqing, China. The waste plastic particles utilized in this study are mainly composed of polypropylene (PP) and various dyeing agents, as shown in Figure 1.

**Figure 2:**
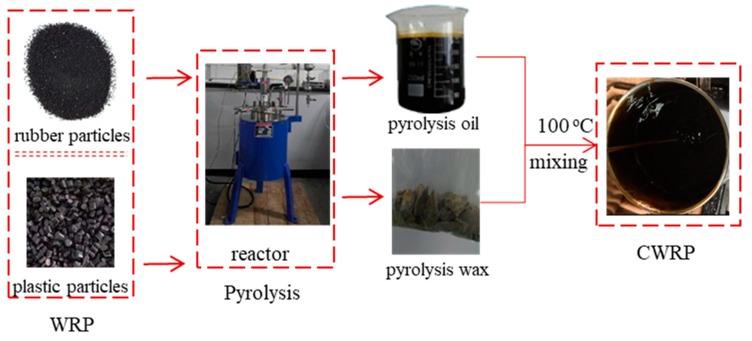
Production program of CWRP.

Figure 2 illustrates the production program of CWRP. As can be seen, pyrolysis oil (PO) and pyrolysis wax (PW) were prepared by pyrolyzing waste rubber and waste plastic, respectively. The PO and PW were then mixed with a weight ratio of 6:1 at 100 °C. 100 °C was set as the mixing temperature of PW and PO as the melting point of PW is around 90 °C.

The CWRP modified asphalt binders (CMA) were prepared by blending CWRP and base binder using a high-speed shear mixer at 115 °C with a rotation speed of 800 rpm for 10 min. The dosage of WRP additive is 5% by weight of base binder. By each blending, around 500 g CMA can be prepared. The mixing temperature of the CMA was determined based on its viscosity–temperature curve.

### 2.2. Experimental Program

The three empirical characteristics—namely, penetration, softening point, and ductility—were employed to evaluate the conventional physical properties of CWRP modified and neat binders. Penetration test were performed at three temperatures (5 °C, 15 °C, and 25 °C) for calculating the penetration index (PI) of binder. The calculated PI was used to characterize the temperature sensitivity of asphalt binders. A Brookfield viscometer was employed to characterize the workability of all types of asphalt binders. The viscosity tests were carried out from 80 °C to 120 °C, with an interval of 10 °C. For each specimen, three replicate tests were conducted. 

A DHR-2 dynamic shear rheometer (DSR, TA Instruments, Inc., New Castle, DE, USA) was utilized to evaluate the rheological performance of asphalt binder. The high temperature performance of asphalt binder was assessed based on the measured rutting factors [66]. Two 20-mm plates with a plate gap of 1 mm were used in the rutting factor test in accordance with ASTM D6373-16 [66]. The rutting factor test was performed on asphalt binders before and after rolling thin film oven (RTFO) aging. Rutting factor was measured under the oscillate load with strain of 12% and 10% for unaged and RTFO aged asphalt binder, respectively. The load frequency was set at 10 rad/s. Testing temperature increases from 52 °C at 6 °C interval until the value of rutting factor equal to or less than 1.0 kPa for unaged binders and 2.2 kPa for RTFO aged binders.

The fatigue property of asphalt binder was characterized using time sweep test [67]. Time sweep tests were carried out at 20 °C under a control–displacement model. The oscillation displacement was set as 6 rad. Repeated sinusoidal load with a constant loading frequency of 10 rad/s was applied on all types of specimens. During the test, complex modulus and phase angles were monitored as a function of loading cycles. For each specimen, three replicate tests were performed.

The low temperature performance was evaluated by Bending Beam Rheometer (BBR) test. This test was performed at a beginning temperature of −12 °C in accordance with ASTM D6648-08 [68]. The test temperature was increased or decreased at 6 °C interval until the conditions (creep stiffness, S ≤ 300 MPa and m-value ≥ 0.300) were reached. Three replicates were prepared and tested.

To evaluate the dispersity of CWRP additive inside neat binder, an XSZ-H FM (Chongqing Optical Instrument Co., Ltd, Chongqing, China) was utilized in this study. FM morphology observation was conducted on the CMA at 100 times magnification. The obtained FM images were further analyzed using MATLAB R2018a software.

To investigate the modification mechanism of CWRP additive, FTIR and DSC were performed on base binder, CWRP and CMA. A Nicolet IS50 FTIR (Nicolet Instrument Technologies, Inc., Madison, WI, USA) with attenuated total reflection (ATR) reflection mode was used to obtain the infrared spectrum. The sample preparation methods of test specimens, as well as the technical parameters of FTIR, were documented in previous studies [9]. DSC Q20 (TA Instruments, Inc., New Castle, DE, USA) was performed under nitrogen atmosphere. Testing temperature increases from −80 °C at a rate of 10 °C per minute until the ended temperature (200 °C) was reached.

## 3. Findings and Discussion

### 3.1. Viscosity

**Figure 3:**
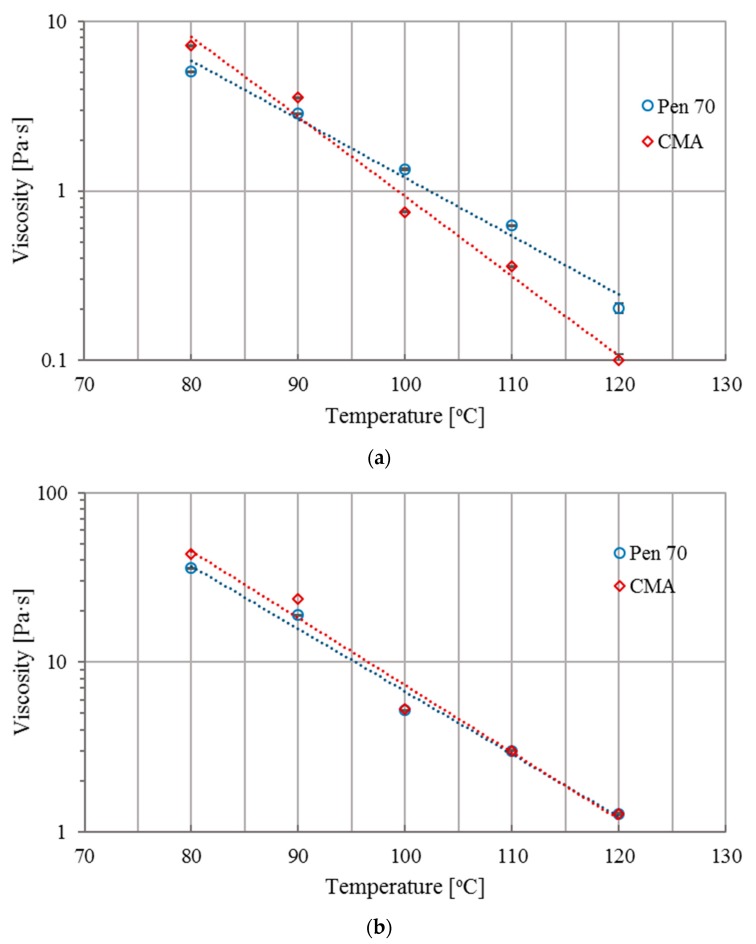
Viscosity of asphalt binders: (**a**) before and (**b**) after RTFO aging.

Viscosity of all asphalt binders from 80 °C to 120 °C were investigated for evaluating the workability of asphalt binders. The viscosity of asphalt binders before and after short-term aging is presented in Figure 3a,b, respectively. It can be seen in Figure 3a that the viscosity of CMA at 115 °C is around 0.17 Pa·s. According to the Superpave mix design manual [69], 115 °C can be set as CMA’s mixing temperature which is 50 °C lower than that of Pen 70. Consequently, CMA was RTFO aged at 120 °C for 85 min for better simulating the short-term aging process [65,70]. In this study, the error bars were drawn based on the standard deviation of the test results.

As shown in Figure 3a, for both base and CWRP modified binder, viscosity declines as the temperature increases, although the decrease rate of CWRP modified binder is higher than that of Pen 70. The decrease rate of CMA sharply increased when temperature was higher than 90 °C, while the decrease rate of Pen 70 remains almost constant. In addition, it is interesting to know that the viscosity of CMA is higher compared to that of Pen 70 if the test temperature is lower than 90 °C. When temperature increases above 90 °C, the viscosity of CMA is much lower than that of Pen 70. This phenomenon is caused by the melting of added PW. When temperature increases above the melting point of PW, PW melts which declines the viscosity of asphalt binder. It indicates that adding CWRP increases the workability of neat binder.

Similarly, the turning point of CMA’s viscosity curve can be observed in the RTFO aged specimens. In addition, data presented in Figure 3b illustrates that within the whole temperature range, the viscosity of short-term aged CMA is higher than that of short-term aged Pen 70 which indicates a better bonding performance of CMA.

### 3.2. Three Empirical Characteristics

**Figure 4:**
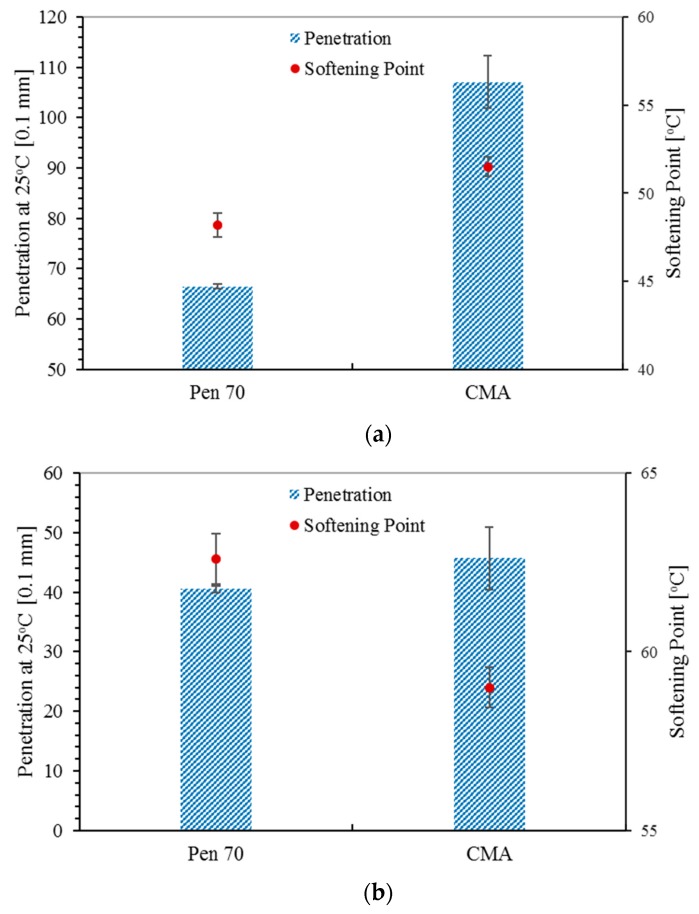
Penetration and softening point of asphalt binders: (**a**) before and (**b**) after RTFO aging.

The results of the penetration (25 °C) and softening point tests are presented in Figure 4.

According to the softening points presented in Figure 4a, it was found that the CWRP modifier increases the softening point of asphalt binder. Like rubber asphalt and other polymer modified asphalt binders, the increasing of softening point could be explained by the swelling and dissolution of the polymers in CWRP. In addition, the wax inside the CWRP also plays a role in increasing the softening point of CMA. After RTFO aging, although the softening point of CMA and Pen 70 are both increased, the softening point of CMA is lower than that of Pen 70. This might be because lighter components in asphalt binder have a strong affinity for CWRP, which is less likely to be volatilized. Consequently, the volatilization of lighter components in asphalt binder during RTFO aging process is weakened. Considering the changes on the properties of asphalt binder resulting from RTFO aging are mainly because of the volatilization of lighter components in asphalt binder, lower volatility leads to a smaller change on penetration value and softening point. Due to the same reason, in comparison with Pen 70, the penetration value of CMA presents a lower sensitivity to RTFO aging.

Based on the penetration values, PI, which is defined by Equations (1) and (2), was calculated for evaluating the temperature sensitivity of asphalt binder. The calculated PIs are listed in Table 2.

(1) $$\mathrm{PI}=\frac{20-500A}{1+50A},$$

(2) lgP=AT+K,

where,

*T* is the temperature at which the penetration test is performed
*P* is the penetration value at the corresponding test temperature
*A* and *K* are determined by the lg (penetration value) vs. temperature curve

PI reveals that the temperature sensitivity of CMA is comparable to that of Pen 70, which means adding CWRP does not negatively affect the temperature sensitivity of binder.

The ductility of CMA at 15 °C is larger than 150 cm while that of Pen 70 is 105.8 cm. This indicates that, in comparison with Pen 70, CMA may have a better low-temperature performance.

### 3.3. High-Temperature Performance

**Figure 5:**
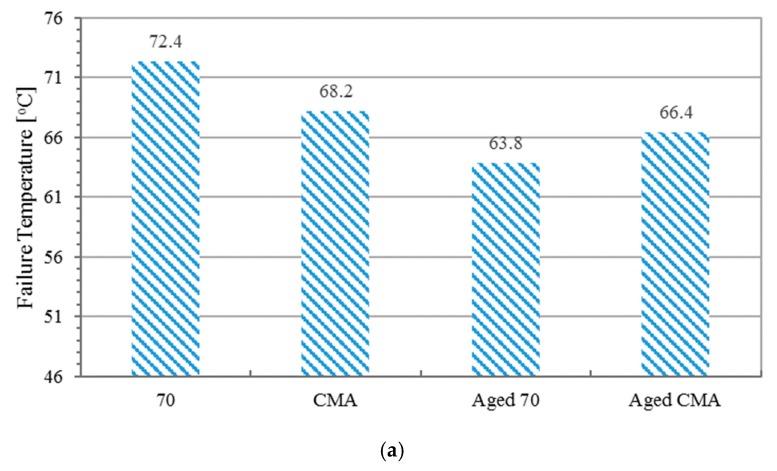

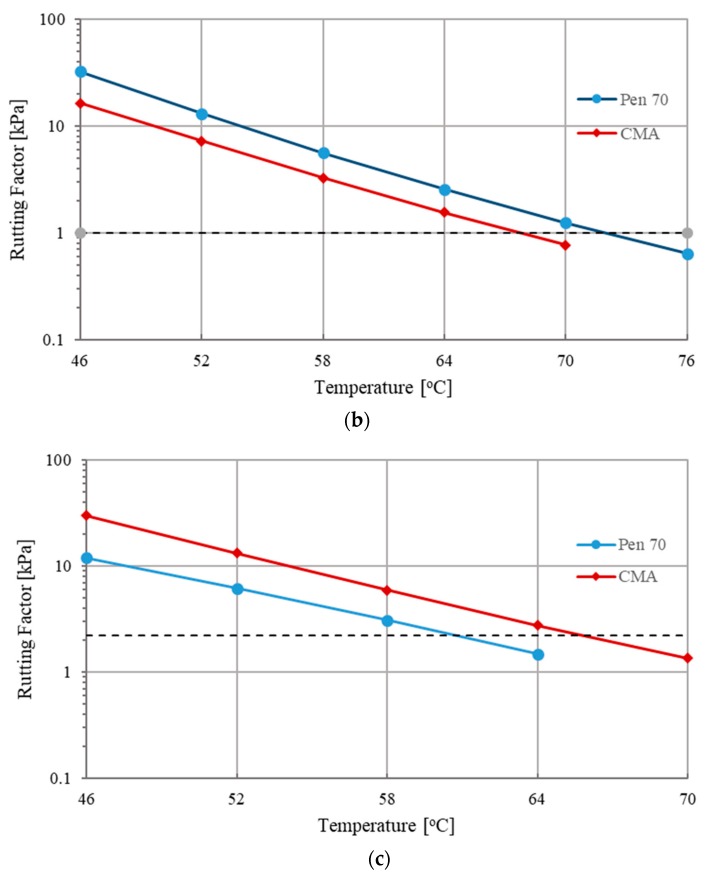
Results of temperature sweep test: (**a**) failure temperature, rutting factor for unaged samples (**b**), and aged samples (**c**).

The high-temperature performance was evaluated by the temperature sweep test. Rutting factors, as well as the failure temperatures, are presented in Figure 5.

As shown in Figure 5a, the failure temperature values of unaged binders are higher than those of aged binders. Before RTFO aging, failure temperature value of neat binder is higher than that of CMA binder. Conversely, after RTFO aging, the failure temperature value of CMA is higher than that of based binder. Consistent with the failure temperature, base binder and CMA show higher rutting factors before and after aging, respectively. Since rutting happened on short-term aged asphalt mixture, CMA shows a better rutting resistance performance.

### 3.4. Low-Temperature Performance

Low temperature performance of both base binder and modified binder were evaluated using both stiffness value and m-value obtained from Bending Beam Rheology (BBR) tests. The test results are listed in Table 3.

According to Table 3, it can be found that the stiffnesses of CMA are much lower than that of Pen 70. While the m-values of CMA are bigger than that of Pen 70. According to the ASTM standard [68], stiffness value should be less than 300 MPa, while the m-value should be larger than 0.3 for a specific temperature grade. Low-temperature cracking is more likely to occur on asphalt binder with higher stiffness. The BBR test results reveals that CWRP significantly increases the low-temperature performance of base binder.

### 3.5. Fatigue Performance

**Figure 6:**
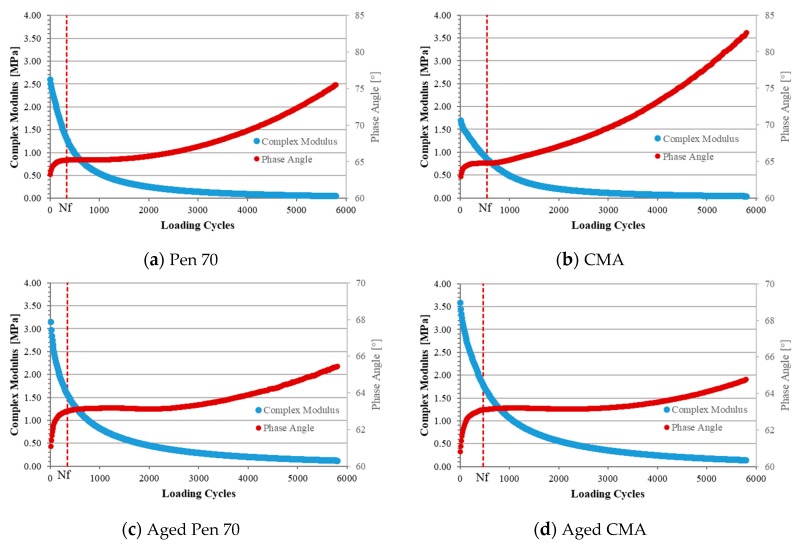
Results of time sweep test: (**a**) Pen 70, (**b**) CMA, (**c**) Aged Pen 70, and (**d**) Aged CMA.

Fatigue performance is evaluated by time sweep test at 25 °C. The results of time sweep test is shown in Figure 6. 50% reduction of initial complex modulus method was utilized to define the fatigue failure point of asphalt binder. The number of failure values (Nf) is presented in Table 4. Higher Nf indicates superior fatigue resistance at the tested strain level.

As can be seen, regardless of the aging state, Nf of CMA is higher than that of Pen 70. It indicates that CMA shows longer fatigue life, which means that CWRP can increase the fatigue life of binder at the selected strain level. 

### 3.6. Dispersity

**Figure 7:**
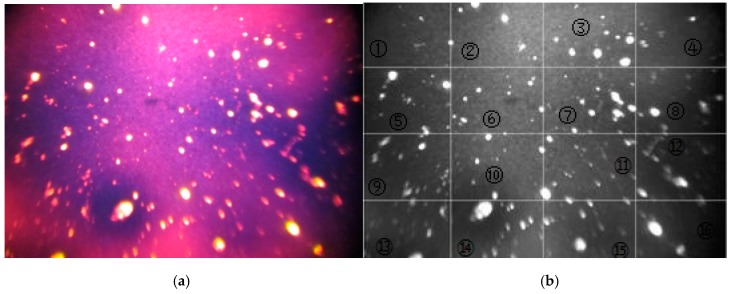
FM image of CWRP modified asphalt binder: (**a**) FM image and (**b**) gridded FM image.

The dispersity is characterized by FM observation providing information on the polymer and insoluble component in the modified binder. Dispersity refers to the degree of fragmentation of the dispersed phase. Dispersity can be characterized by evaluating the average diameter of dispersed phase. FM observed information was analyzed using MATLAB to calculate the number of particles and the corresponding diameters. Figure 7 shows the FM image of CWRP modified asphalt binder.

As shown, the FM image was gridded into 16 sections. In each section, particle numbers and particle diameters were collected from the FM image. The average diameters were then calculated based on the collected particle number and particle diameter. Particle numbers in each section and the corresponding average diameters were listed in Table 5.

To evaluate the differences among particle numbers and average diameter, normality tests and outlier test were performed in advance. Kolmogorov-Smirnov (K-S) testing was performed on both the particular number and average diameter. The K-S test results are shown in Table 6.

As can be seen, for both particular number and average diameter, the Asymptotic Significance (Asymp. Sig.) is larger than 0.05 which indicates that the distribution of the particular number and average diameter is normal.

**Figure 8:**
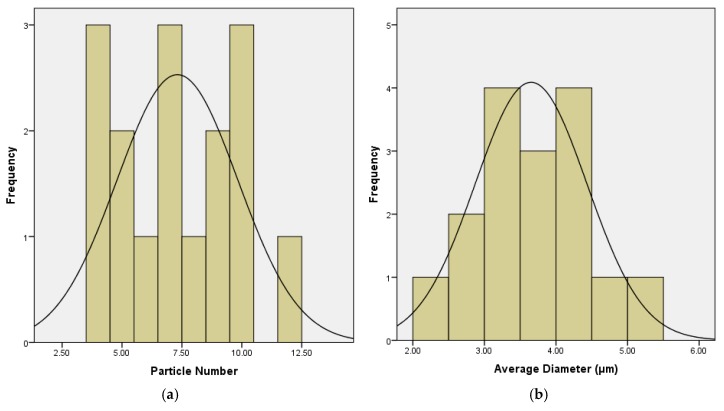
Results of normality test: (**a**) particle number and (**b**) average diameter.

**Figure 9:**
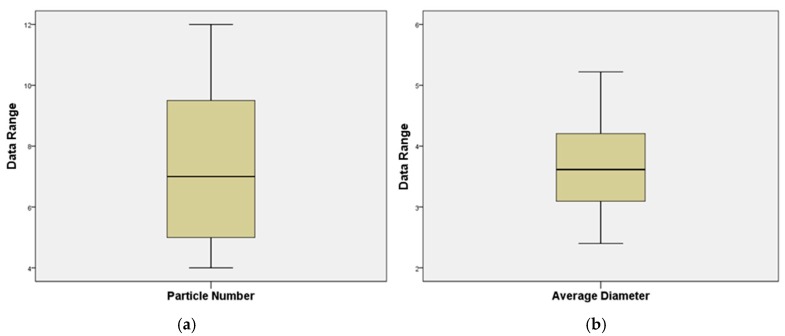
Results of outlier test: (**a**) particle number and (**b**) average diameter.

The results of normality testing and outlier testing are presented in Figure 8 and Figure 9, respectively. As expected, both particle number and average diameter follow the normal distribution with the mean value of 7.31 and 3.65, respectively.

Box plot was employed to detect the outliers of the data set. The upper and lower limit of the non-outlier were determined based on the quantile and an empirical constant K (K = 1.5). Specifically, if Q_1_ and Q_3_ are the lower and upper quartiles, respectively, then the non-outlier range is defined as [Q_1_ − k(Q_3_ − Q_1_), Q_3_ + k(Q_3_ − Q_1_)]. Data out of the range is defined as outliers. As shown in Figure 9, since all the data were observed within the non-outlier range, it can be concluded that no outliers were detected in these two data sets.

To further analyze the difference among the particle number and average diameter in each section, the sections were divided into eight groups. Section 1 and 2 are group 1, section 3 and 4 are group 2, and so on. ANOVA testing was then performed at a 95% confidence interval for determining the relationship between group and particle number and average diameter. The ANOVA test results are shown in Table 7. This table illustrates that for both particle number and average diameter, no significant difference between groups can be detected, which means the dispersity of CWRP inside base binder is statistically uniform.

### 3.7. Fourier Transform Infrared Spectroscopy

**Figure 10:**
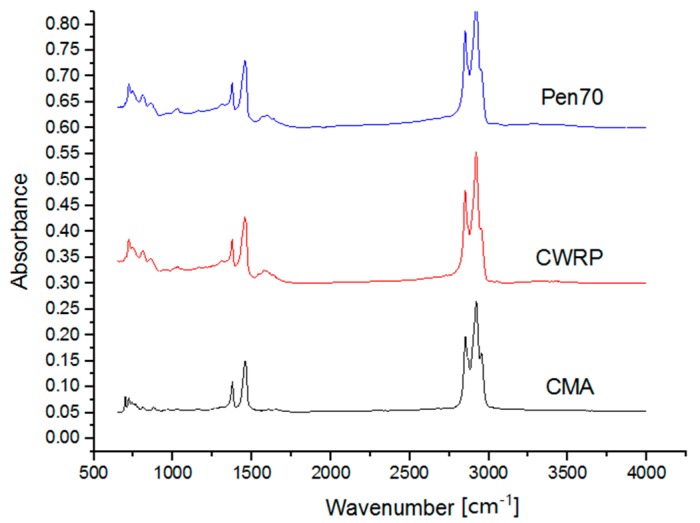
FTIR test results.

FTIR tests were performed on both asphalt binders and the additive for determining the effects of CWRP additive on the chemical composition of base binder. Figure 10 illustrates the FTIR spectrum of Pen 70 base binder, CWRP modifier, and CWRP modified asphalt binder.

In these spectrums, the major bands around 2920 cm^−1^ and 2852 cm^−1^ resulted from the vibration of stretching vibrations of Alkyl C–H and Aliphatic C–H, respectively. The absorption at around 1456.50 cm^−1^ and 1376.47 cm^−1^ are caused by the blending vibration of methylene and methyl, respectively, while those at 720.78 cm^−1^ and 698.12 cm^−1^ are due to the plane swing vibration of the methylene in alkyl group.

As can be seen, both the binders and modifier have the absorption peaks at same wavenumbers, so no major chemical reactions were detected due to the addition of CWRP modifier. 

### 3.8. Differential Scanning Calorimetry

**Figure 11:**
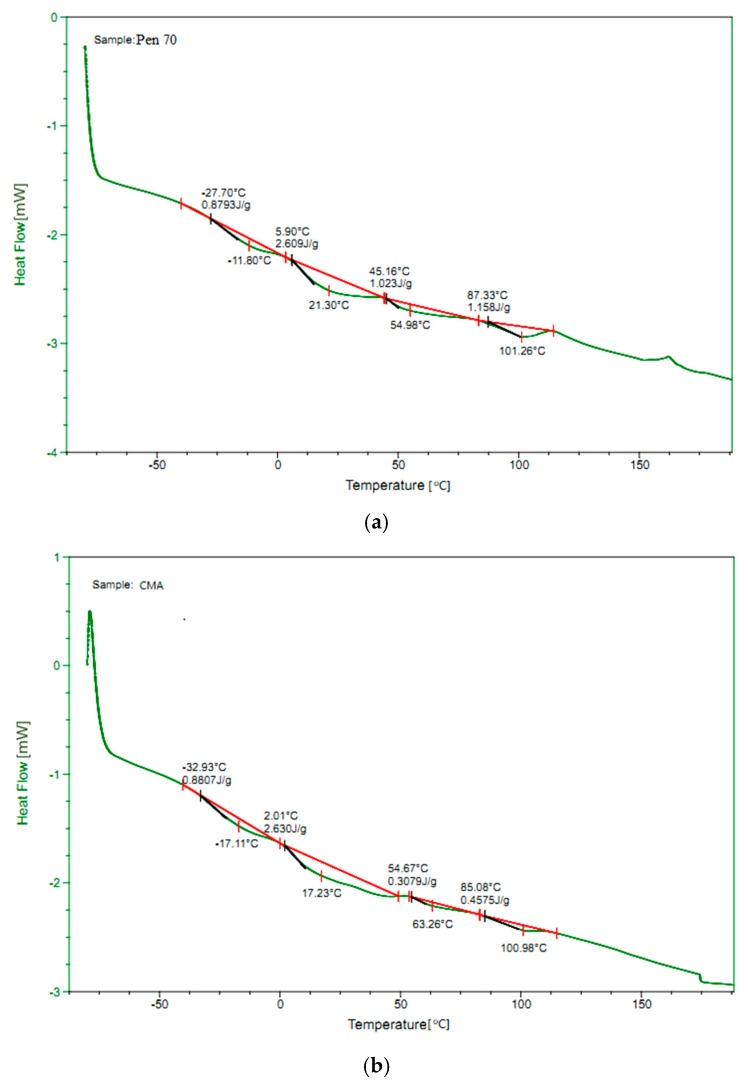
Result of DSC tests: (**a**) Pen 70 and (**b**) CMA.

Differential scanning calorimetry (DSC) is a thermos-analytical method that allows the determination of physical changes in a material associated with a heat exchange. The properties that are especially relevant for bitumen are physical changes such as glass transition temperature (Tg) and phase transition such as melting and crystallization. The DSC tests were carried out to characterize the thermal property of all the asphalt binders. Figure 11 shows the DSC results.

Previous studies demonstrated that lower Tg indicates better low-temperature performance, and vice versa [71,72]. Thus, Tg were utilized to evaluate the low temperature of asphalt binder. As can be seen, the Tg of CMA is around 5.3 °C lower than that of Pen 70. Again, it indicates that CWRP have positive influence on the low-temperature performance of asphalt binder. In addition, around the softening point of asphalt binder, the exothermic peak of CMA occurred few degrees later than that of Pen 70 which reveals the adding CWRP also increases the high-temperature performance of asphalt binder. In addition, no significant differences can be found between the DSC curve of CMA and Pen 70. By combining with the results of FTIR, it can be proposed that the modification mechanism of CWRP is mainly because of the physical change instead of chemical reaction.

## 4. Findings and Recommendations

This paper presents a laboratory study to characterize and compare the rheological properties of Pen 70 modified with CWRP. According to the test results on viscosity, rutting factors, time sweep, BBR test, FM test, FTIR, and DSC, the following major findings have been obtained:

- The incorporation of CWRP additives enhances the rutting resistance, low-temperature, and fatigue life of virgin asphalt binder. The enhancement comes from the modification effect by the polymers in CWRP.
- Adding CWRP increases the workability because of the lighter compositions and wax in CWRP.
- CWRP is found to be uniformly distributed inside asphalt binder.
- Chemical reaction between CWRP and based binder cannot be detected. The modification of CWRP is more likely due to physical change.

Based on the limited findings of this study, recycling WRP into asphalt binder towards enhanced mechanical properties and environmental benefits appears promising. CWRP has been found to be able to increase the workability and service performance of asphalt binder. Further research on the performance of asphalt mixture is required. In addition, adjusting the thermal cracking process for producing asphalt binder modifier with better performance is also recommended. 

## Figures and Tables

**Table 1:** Properties of Pen 70 asphalt binder.

Description	Units	Results	Methods
Penetration at 25 °C	0.1 mm	68	ASTM D5 [57]
Softening point	°C	51.4	ASTM D36/D36M [58]
Dynamic viscosity at 60 °C	Pa∙s	226	ASTM D4402/D4402M [59]
Ductility at 15 °C	cm	>100	ASTM D113 [60]
Wax content	%	0.7	DIN EN 12606 [61]
Flash point	°C	283	ASTM D92 [62]
Density at 15 °C	g/cm^3^	1.03	ASTM D70 [63]
Solubility in trichloroethylene	% (by weight)	99.8	ASTM D2042 [64]
Rolling thin film oven test at 163 °C for 85 min			ASTM D2872 [65]
Penetration of residue at 25 °C	%	51.68	ASTM D5 [57]
Ductility of residue at 10 °C	cm	11	ASTM D4402/D4402M [59]

**Table 2:** PI of asphalt binder.

Binder Type	PI
Pen 70	−1.2
CMA	−1.4

**Table 3:** Results of BBR test.

Temperature (°C)	Binder Type	Stiffness Value (MPa)	m-Value
−12	Pen 70	98	0.42
	CMA	not applicable *	0.58
−8	Pen 70	230	0.35
	CMA	48	0.50
−24	Pen 70	353	0.21
	CMA	148	0.40

* The stiffness value of CMA at −12 °C over the measurement capacity of BBR.

**Table 4:** Number of failure values.

Binder Type	Aging State	Fatigue Life (cycles)
Pen 70	Unaged	337
	RTFO aged	464
CMA	Unaged	340
	RTFO aged	547

**Table 5:** Particle number and average diameter in each section.

Section No.	Particle Number	Average Diameter (µm)	Standard Deviation
1	4	2.69	0.570903
2	7	3.08	
3	10	3.64	
4	5	3.76	
5	9	3.26	
6	10	2.4	
7	10	3.59	
8	5	4.46	
9	7	4.12	
10	8	2.71	
11	9	3.37	
12	6	4.29	
13	4	4.62	
14	12	3.11	
15	7	4.11	
16	4	5.22	

**Table 6:** Results of Kolmogorov–Smirnov test.

Test Item		Particular Number	Average Diameter
N		16	16
Normal Parameters	Mean	7.3125	3.6519
	Standard Deviation	2.52240	0.78036
Most Extreme Differences	Absolute	0.133	0.096
	Positive	0.133	0.079
	Negative	−0.123	−0.096
Test Statistic		0.133	0.096
Asymptotic Significance (two-tailed)		0.200	0.200

**Table 7:** Results of ANOVA test.

Test Item	F	Sig.
Particle number	0.383	0.888
Average diameter	1.464	0.301
